# Reticulated violaceous patches in a 10-year-old boy

**DOI:** 10.1016/j.jdcr.2024.09.020

**Published:** 2024-10-11

**Authors:** Cassidy M. Nguyen, Emily Clarke, Rosemary Peterson, Brett H. Keeling, Lucia Z. Diaz

**Affiliations:** a Division of Dermatology, Department of Internal Medicine, Dell Medical School, University of Texas at Austin, Austin, Texas; b Division of Pediatric Rheumatology, Department of Pediatrics, Dell Medical School, University of Texas at Austin, Austin, Texas; c Dell Children’s Medical Center, Austin, Texas; d Division of Pediatric Dermatology, Department of Pediatrics, Dell Medical School, University of Texas at Austin, Austin, Texas

**Keywords:** adenosine deaminase 2 deficiency, DADA2, livedo racemosa, livedo reticularis, polyarteritis nodosa

## Patient history

**Fig 1:**
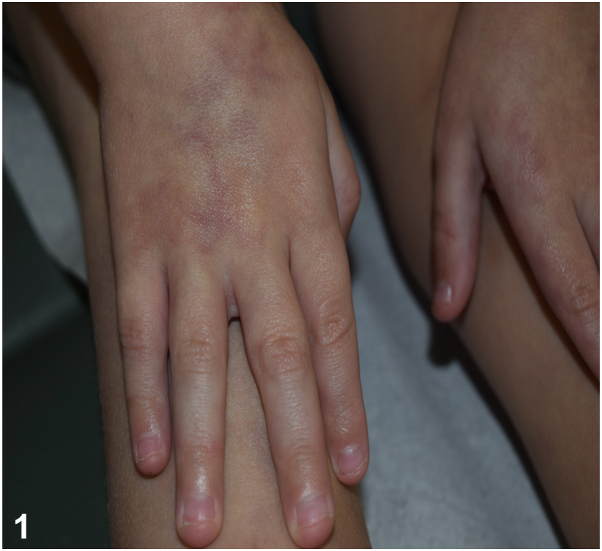

**Fig 2:**
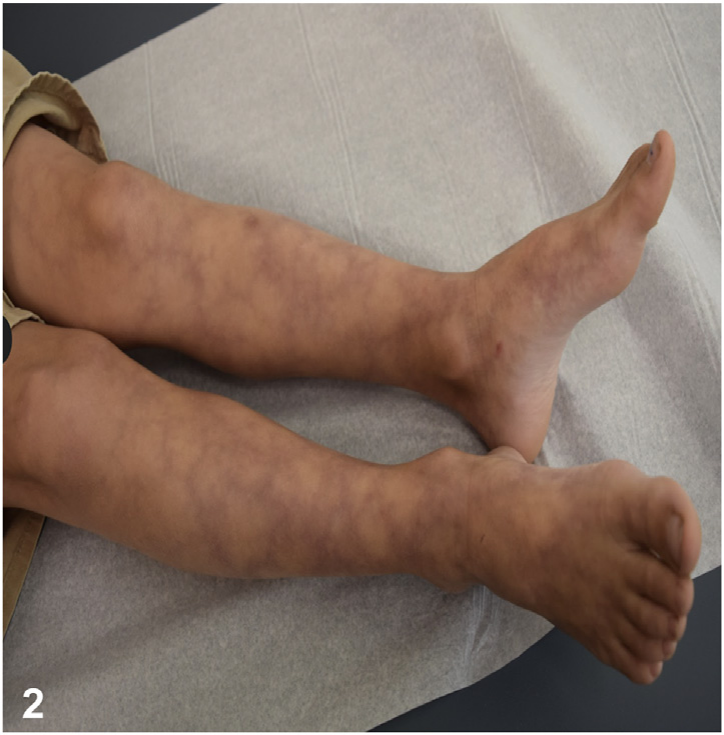

**Fig 3:**
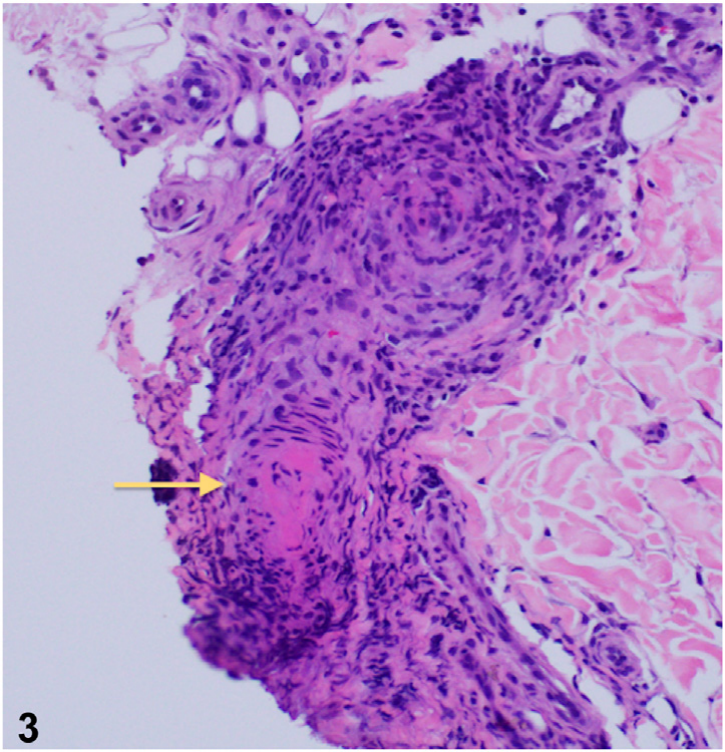

A 10-year-old healthy boy presented for a generalized asymptomatic skin eruption for 6 years. Examination was notable for reticulated violaceous patches on the trunk and extremities (Figs 1 and 2). A punch biopsy was taken from an affected area on the right leg (Fig 3). His laboratory workup included a normal complete blood count, antinuclear antibody test, immunoglobulin levels, and lymphocyte subsets. He had an elevated c-reactive protein 6.4 mg/L and a normal comprehensive metabolic panel except for an elevated alanine transaminase 58 U/L. Additionally, his adenosine deaminase (ADA) enzyme activity was low at 1.2 μ/mL (normal 4.3-11.4 μ/mL). The patient’s past medical history did not include any serious infections or hospitalizations.

**Question 1: Which of the following is the most likely diagnosis?**

A. Deficiency of adenosine deaminase 2 (DADA2)
B. IgA vasculitis
C. Severe combined immune deficiency syndrome
D. Cutis marmorata telangiectatica congenita
E. Capillary malformation-arteriovenous malformation syndrome

**Answers:**

A. Deficiency of adenosine deaminase 2 (DADA2) – Correct. DADA2 is an autosomal recessive autoinflammatory disease characterized by systemic vasculitis, early-onset stroke, bone marrow failure, and immunodeficiency. This rare condition is caused by loss-of-function pathogenic variants in the ADA2 gene and is definitively diagnosed by measurement of plasma or serum ADA2 enzymatic activity and/or sequencing of the ADA2 gene.^1^ Biopsy, as above, may demonstrate necrotizing inflammation of a medium-sized arteriole with intraluminal thrombus suggestive of a vasculitic etiology.
B. IgA vasculitis – Incorrect. IgA vasculitis also known as Henoch-Schönlein purpura, is a small-vessel vasculitis that predominantly occurs in children. Patients usually present with palpable purpura that may become necrotic. These lesions often appear on the bilateral lower extremities and buttocks, but can occur anywhere on the body. This may also be associated with arthritis and renal involvement.
C. Severe combined immune deficiency syndrome – Incorrect. Severe combined immune deficiency syndrome can also have low ADA enzyme activity, but patients are often susceptible to severe infections, and the patient’s immune workup in this case was reassuring. Cutaneous manifestations often appear more eczematous if skin changes are present.
D. Cutis marmorata telangiectatica congenita – Incorrect. This condition presents at birth with violaceous, reticular patches on the lower extremities that may be associated with focal atrophy, telangiectasias, and ulcerations. These often improve with time, but rarely disappear, and they do not resolve with warming such as cutis marmorata. Patients who have generalized involvement may have associated abnormalities including neurologic, cardiac, vascular, and musculoskeletal anomalies.
E. Capillary malformation-arteriovenous malformation syndrome – Incorrect. This is a vasculitic syndromic disorder caused by a mutation in the *RASA1* gene that is characterized by superficial capillary and arteriovenous malformations that present as erythematous to violaceous patches. Lesions may present with peripheral, blanching halos, are warm to the touch, and can be auscultated with a thrill or bruit heard on examination.

**Question 2: Which of the following manifestations are associated with this disease?**

A. Livedo racemosa
B. Early-onset hemorrhagic and ischemic strokes
C. Arthralgia and arthritis
D. Uveitis
E. All of the above

**Answers:**

A. Livedo racemosa – Incorrect. Livedo racemosa is a common cutaneous manifestation in DADA2.^2^
B. Early-onset hemorrhagic and ischemic strokes – Incorrect. One of the most concerning features of DADA2 is the risk of early-onset ischemic and hemorrhagic stroke. As such it is important to consider initiation of treatment of tumor necrosis factor inhibitor (TNFi) even if the patient is asymptomatic given the mortality rate is 8% before the age of 30 years.^1^
C. Arthralgia and arthritis – Incorrect. Musculoskeletal manifestations occur in 40% of affected individuals. Patients affected by myalgia/arthralgia (22%) and arthritis (14%) also have concurrent signs of systemic inflammation including elevated acute-phase reactants and fevers.^3^
D. Uveitis – Incorrect. Uveitis may be seen in patients with DADA2, along with vision loss, diplopia, retinal infarcts, optic nerve damages, ptosis, strabismus, and nystagmus.^3^
E. All of the above – Correct. Patients with DADA2 may have variable clinical presentation, which include cutaneous, neurologic, ophthalmologic, and musculoskeletal findings.

**Question 3: Which of the following is recommended as first-line long-term treatment?**

A. Hematopoietic stem cell transplantation
B. TNF-α inhibitors
C. Steroids
D. Thalidomide
E. No treatment

**Answers:**

A. Hematopoietic stem cell transplantation – Incorrect. Although it can be used for rescue therapy in patients with severe hematologic and immunologic disorders, it is associated with severe adverse effects. TNF-α inhibitors is first-line treatment for DADA2 given the reduction in cerebrovascular events on this treatment.^1^
B. TNF-α inhibitors – Correct. Although no defined treatment protocols exist, studies have repeatedly shown the importance of TNFi therapy to mitigate the central nervous system sequelae, including risk of stroke, in both symptomatic and asymptomatic patients with DADA2.^1^ Current TNFi include the soluble anti-TNF receptor etanercept and monoclonal antibodies adalimumab, infliximab, and golimumab. Because of the risk of cerebrovascular events, TNFi therapy is indefinite. TNFi discontinuation and suboptimal dosing have been associated with disease flares.^1^
C. Steroids – Incorrect. Immunosuppressive medications including steroids, azathioprine, cyclosporine, cyclophosphamide, methotrexate, mycophenolate mofetil, and tacrolimus may be used for short-term flares. However, these have variable success in the treatment of DADA2. TNFi is required for long-term treatment for the prevention of strokes.^3^
D. Thalidomide – Incorrect. Although thalidomide can inhibit TNF production and studies have shown complete response to treatment, adverse side effects including sensory peripheral neuropathy, such as paresthesias, tremors, and dizziness have been reported.^3^
E. No treatment – Incorrect. Without treatment, DADA2 has an estimated 8% mortality rate before 30 years old, making prompt diagnosis and appropriate management paramount.^1^ Because the risk of stroke can be mitigated with TNFi, even asymptomatic patients should consider treatment.

## Conflicts of interest

None disclosed.

## References

[1] Lee P.Y., Davidson B.A., Abraham R.S. (2023). Evaluation and management of deficiency of adenosine deaminase 2: an international consensus statement. JAMA Netw Open.

[2] Chasset F., Fayand A., Moguelet P. (2020). Clinical and pathological dermatological features of deficiency of adenosine deaminase 2: a multicenter, retrospective, observational study. J Am Acad Dermatol.

[3] Kasap Cuceoglu M., Sener S., Batu E.D. (2021). Systematic review of childhood-onset polyarteritis nodosa and DADA2. Semin Arthritis Rheum.

